# Statement of Retraction: Circular RNA_0120376 regulates microRNA-148b-3 and centrosomal protein 55 to promote non-small cell lung cancer development

**DOI:** 10.1080/21655979.2026.2728875

**Published:** 2026-10-01

We, the Editors and Publisher of the journal *Bioengineered*, have retracted the following article from publication.

Du, T., Yi, S., Wang, Y., Zhao, Q., Ma, P., & Jiang, W. (2022). Circular RNA_0120376 regulates microRNA-148b-3 and centrosomal protein 55 to promote non-small cell lung cancer development. *Bioengineered*, 13(5), 11844–11855. https://doi.org/10.1080/21655979.2022.2052647

Following publication, internal integrity audits revealed concerns around several authorship changes which were made between submission and acceptance of the article. In addition, discrepancies were noted in the ethics statements included in the article.

We have contacted the authors for an explanation, but we have not received a response. As determining authorship and contributions and ensuring the ethical conduct of the research are core to the integrity and accountability of published work, we are therefore retracting the article. The authors have been informed of this decision.

We have been informed in our decision-making by our policy on publishing ethics and integrity and COPE guidelines.

The retracted article will remain online to maintain the scholarly record and will be digitally watermarked on each page as ‘Retracted’.

